# ^1^H NMR-Based Metabolomics Approach Revealing Metabolite Variation of Black Turmeric (*Curcuma caesia*) Extracts and Correlation with Its Antioxidant and α-Glucosidase Inhibitory Activities

**DOI:** 10.17113/ftb.61.01.23.7711

**Published:** 2023-03

**Authors:** Nurul Najiha Ain Ibrahim, Nurkhalida Kamal, Ahmed Mediani, Azliana Abu Bakar Sajak, Soo Yee Lee, Khozirah Shaari, Hafeedza Abdul Rahman

**Affiliations:** 1 Department of Food Sciences, Faculty of Science and Technology, Universiti Kebangsaan Malaysia, 43600 UKM Bangi, Selangor, Malaysia; 2 Institute of Systems Biology (INBIOSIS), Universiti Kebangsaan Malaysia, 43600 UKM Bangi, Selangor, Malaysia; 3 Department of Nutrition and Dietetics, Faculty of Medicine and Health Sciences, Universiti Putra Malaysia, 43400 UPM Serdang, Selangor, Malaysia; 4 Current address: Clinical Research Centre, Division of Medical Education and Research, Sunway Medical Centre Sdn. Bhd, Selangor, Malaysia; 5 Laboratory of Natural Medicines and Products (NaturMeds) Institute of Bioscience, Universiti Putra Malaysia, 43400 Serdang, Selangor, Malaysia; 6 Current address: School of Food Studies & Gastronomy, Faculty of Social Sciences & Leisure Management, Taylor's University, Subang Jaya, 47500, Malaysia; 7 Current address: Food Security & Nutrition Impact Lab, Taylor’s University, Subang Jaya, 47500, Selangor, Malaysia; 8 Centre of Excellence, Innovation Centre for Confectionery Technology (MANIS), Faculty of Science and Technology, Universiti Kebangsaan Malaysia, 43600 UKM Bangi, Selangor, Malaysia

**Keywords:** antioxidant activity, α-glucosidase inhibition, *Curcuma caesia*, extraction, metabolomics

## Abstract

**Research background:**

*Curcuma* species (Zingiberaceae) are well known medicinal herbs in India and Southeast Asia. Despite various findings reporting their beneficial biological activities, very little information has been recorded on the *Curcuma caesia*. Thus, this study aims to determine the phenolic content, antioxidant and α-glucosidase inhibitory activity of both rhizome and leaves of *C. caesia*.

**Experimental approach:**

Rhizome and leaves of *C. caesia* were dried with oven (OD) and freeze (FD)-drying methods, and extracted with different *Φ*(ethanol,water)=100:0, 80:20, 50:50 and 0:100. The bioactivities of *C. caesia* extracts were evaluated using *in vitro* tests; total phenolic content (TPC), antioxidant (DPPH and FRAP) and α-glucosidase inhibitory activity. Proton nuclear magnetic resonance (^1^H NMR)-based metabolomics approach was employed to differentiate the most active extracts based on their metabolite profiles and correlation with bioactivities.

**Results and conclusions:**

The FD rhizome extracted with *Φ*(ethanol,water)=100:0 was observed to have potent TPC expressed as gallic acid equivalents, FRAP expressed as Trolox equivalents and α-glucosidase inhibitory activity with values of (45.4±2.1) mg/g extract, (147.7±8.3) mg/g extract and (265.5±38.6) µg/mL (IC_50_), respectively. Meanwhile, for DPPH scavenging activity, the *Φ*(ethanol,water)=80:20 and 100:0 extracts of FD rhizome showed the highest activity with no significant difference between them. Hence, the FD rhizome extracts were selected for further metabolomics analysis. Principal component analysis (PCA) showed clear discrimination among the different extracts. Partial least square (PLS) analysis showed positive correlations of the metabolites, including xanthorrhizol derivative, 1-hydroxy-1,7-bis(4-hydroxy-3-methoxyphenyl)-(6*E*)-6-heptene-3,4-dione, valine, luteolin, zedoardiol, β-turmerone, selina-4(15),7(11)-dien-8-one, zedoalactone B and germacrone, with the antioxidant and α-glucosidase inhibition activities, whereas curdione and 1-(4-hydroxy-3,5-dimethoxyphenyl)-7-(4-hydroxy-3-methoxyphenyl)-(l*E,*6*E*)-1,6-heptadiene3,4-dione were correlated with α-glucosidase inhibitory activity.

**Novelty and scientific contribution:**

*C. caesia* rhizome and leaf extracts contained phenolic compounds and had varies antioxidant and α-glucosidase inhibitory capacities. These findings strongly suggest that the rhizomes of *C. caesia* are an invaluable natural source of active ingredients for applications in pharmaceutical and food industries.

## INTRODUCTION

Plants have been exploited for various purposes including in treating health problems. According to WHO, more than 80% of the world population uses herbal plants for combating diseases. Zingiberaceae family comprises more than 80 different species of rhizomatous herbs, including *Curcuma caesia*. It is known as black turmeric and is mostly native to tropical South Asia. The plant is used traditionally to treat various ailments including asthma, bronchitis, cough, cancer, epilepsy, fever, wounds, leucoderma, pneumonia, cold, piles, tumour, toothache, vomiting and gout (*1*, *2*). Several studies conducted on *C. caesia* revealed its bioactivities, such as antibacterial, antimicrobial, antifungal (*3*), antitumour, antiulcer (*4*), anticancer, antithrombolytic (*5*), antioxidant, anti-inflammatory and analgesic (*6*, *7*). Moreover, this plant is discovered to contain steroids, phenols, alkaloids, flavonoids and tannins (*8*), as well as essential oils (*9*, *10*). These metabolites might be correlated with the plant medicinal properties. Several studies have been performed on the bioactivities of *C. caesia*, however findings on the metabolites responsible for their pharmacological activities are still scarce.

Metabolomics has emerged as an advanced technology tool, which involves quantitative and qualitative assessments of small molecules known as metabolites. It has diverse applications in various science fields, including pharmacology, drug discovery, toxicology, nutrition and crop improvement, using samples ranged from plant and food to human and animal biofluids or tissues (*11*). In metabolomic studies, metabolic profiles can be acquired through high-throughput metabolic analysis using technologies, such as liquid chromatography-mass spectrometry (LC-MS), gas chromatography-mass spectrometry (GC-MS) and nuclear magnetic resonance (NMR) followed by multivariate data analysis (MVDA) for data mining (*12*). Among the various analytical platforms, proton NMR (^1^H NMR) is a popular choice due to its durability, repeatability, non-destructive nature, and easy sample preparation. In addition, the large number of compounds that have been identified lead to the emergence of ^1^H NMR as a primary analytical tool in metabolomic studies (*13*). In the study of medicinal plants, the combination of ^1^H NMR and MVDA has been used to determine the phytochemical changes of plant extracts resulted from different processing steps and their relationship with the studied bioactivity (*14*, *15*).

Plant metabolites are made up of non-polar and polar compounds that play essential roles in preventing oxidative stress and acting as free radical scavengers which are responsible for the development of degenerative diseases such as cancers and diabetics (*16*). For the development of plant (herb)-based nutraceutical products, these valuable compounds should be extracted efficiently. Factors such as drying techniques, extraction method, parameters during extraction such as solvent, temperature and time, particle size of raw materials and solid-to-liquid ratio are among those affecting the extraction efficiency (*17*). Efficacy of extraction affects the amount and types of extracted metabolites from the plant matrix, which reflects the plant extract’s bioactivities. Therefore, the optimizing of the combination of drying techniques and extraction solvents is needed to ensure maximum bioactive metabolites were extracted from the samples.

In this research, the antioxidant and α-glucosidase inhibition activities of rhizomes and leaves of *C. caesia* dried with different drying techniques, freeze-drying (FD) and oven drying (OD), and extracted with different ethanol to water ratios (*Φ*(ethanol,water)=0:100, 50:50, 80:20 and 100/0) were measured. The most potential and promising extract in terms of bioactivity were then analyzed using ^1^H NMR-based metabolomic approach for the chemical profiles. In addition, the correlations between metabolites and the bioactivities (DPPH, FRAP and α-glucosidase) were also determined by applying different MVDA tools. Thus, the outcome of this study may enhance the potential value of *C. caesia* and provide useful information for further research.

## MATERIALS AND METHODS

### Chemicals

All chemicals used in this research are of analytical grade. The chemical used were Folin-Ciocalteu phenol reagent (Merck, Darmstadt, Germany), sodium carbonate, gallic acid, 2,2-diphenyl-1-picrylhydrazyl (DPPH), ethanol, methanol, ascorbic acid, butylated hydroxyanisole (BHA), iron(III) chloride hexahydrate (Merck, Darmstadt, Germany), 2,4,6-Tris (2-pyridyl)-*s*-triazine (TPTZ), hydrochloric acid, sodium acetate trihydrate, glacial acetic acid (Merck, Darmstadt, Germany), Trolox, α-glucosidase (Megazyme, Sydney, Australia), *p*-nitrophenyl-α-glucopyranoside (*p*-NPG substrate), disodium hydrogen phosphate, sodium dihydrogen phosphate, glycine, and quercetin. For NMR, chemicals used were potassium dihydrogen phosphate, deuterated methanol-d_4_ (CD_3_OD), deuterium oxide (D_2_O), sodium deuteroxide (NaOD) (Cambridge Isotope Laboratories, Tewksbury, USA), 3-(trimethylsilyl)propionic acid-d_4_ sodium salt (TSP) (Acros Organic, Geel, Belgium). All other non-stated source of chemicals was from Sigma-Aldrich (St. Louis, MO, USA).

### Plant materials

**Fig. S1:**
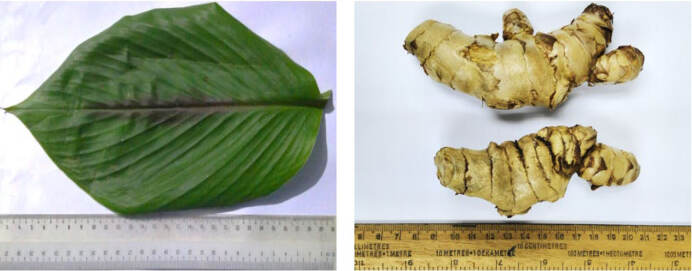
Leaf (left) and rhizome (right) of *Curcuma caesia*

Plant samples (Fig. S1) were collected from Seri Subuh Herbal Park located in Kuala Pilah, Negeri Sembilan, Malaysia. The plant identification and certification were performed by botanists from the Herbarium research unit, Universiti Kebangsaan Malaysia (UKM) with specimen number of UKMB40386.

### Drying process

After collection, samples were rinsed with water to remove debris. The rhizome and leaf samples were labelled and separated into two groups, which were FD and OD. The FD sample was prepared by subjecting it to freezing (HVF-301S Hesstar, Selangor, Malaysia) at −20 °C for 24 h and then lyophilization in a freeze dryer (Freeze Dryer Alpha 1-2 LD Plus, Osterode, Germany). Meanwhile for OD, sample was placed in the laboratory oven at 40 °C (UM400, Memmert Universal Ovens, Schwabach, Germany) under forced-air ventilation. The grinding was for the dried samples to a fine powder using a laboratory grinder (Waring Blender 7011S, Torrington, USA) and sieving was done using a 300 µm siever (mesh size: No. 50).

### Sample extraction

Sample extraction was performed with some modifications (*18*). Ethanol and water were used as extraction solvents at different volume ratios: *Φ*(ethanol,water)=100:0, 80:20, 50:50 and 0:100. The dried powder (20 g) of each rhizome and leaf was immersed in 200 mL of solvent and left for 72 h in an incubator shaker (Ika Control Cooled Incubator Shaker KS4000, Wilmington, NC, USA) at ambient temperature 25 °C. Next, the filtration was performed using Whatman filter paper No. 1 and this step was repeated three times every 24 h. The filtered solvent was then combined before being removed using a rotary evaporator at 40 °C. The resulting viscose samples were frozen at -20 °C overnight and dried in a freeze dryer to remove excess water. All of the extracts were dissolved into the concentration required for the use of chemical analysis.

### Total phenolic content assay

The total phenolic content (TPC) of *C. caesia* extracts was determined by using method described in the previous literature with slight modification (*18*). Each sample (20 µL) with concentration 5000 µg/mL was transferred into a 96-well microplate, followed by 100 µL Folin-Ciocalteu reagent and left to rest for 8 min. Later, 80 µL of 7.0% Na_2_CO_3_ solution was added into the mixture. The resulting mixture was shaken before incubated in the dark for 60 min at room temperature. The absorption was measured with the spectrophotometer (Epoch^TM^ Microplate Spectrophotometer, Biotek Instrument, Santa Clara, CA, USA) at *λ*=725 nm when blue complex was formed. The same procedure was repeated for the standard gallic acid solution and the calibration curve was obtained as follows:

y=0.0057x+0.147, R^2^=0.9982 /1/

Based on the absorbance readings, the TPC of the sample was calculated from the calibration curve and results are expressed as milligrams gallic acid equivalents per gram extract.

### DPPH radical scavenging assay

DPPH radical scavenging assay was examined and modified from the method described in the literature (*18*). Briefly, 250 μL of methanolic 0.1 mM DPPH solution was added to 50 μL sample extracts (78.13–5000 µg/mL) in a 96-well microplate. The mixtures were allowed to be in the dark for 30 min at room temperature. After 30 min, the absorption was measured at *λ*=517 nm with Epoch™ microplate spectrophotometer (Biotek Instrument) against a blank containing 250 μL of methanol. The percentage of DPPH scavenging activity was evaluated using the formula:

Scavenging activity=((*A*_b_–*A*_s_)/*A*_b_)∙100 /2/

where *A*_b_ is the blank absorbance and *A*_s_ is the sample absorbance. The procedure was repeated by replacing the sample with ascorbic acid (AA) and butylated hydroxyanisole (BHA) as positive controls. The results were expressed as IC_50_ in µg/mL, which was obtained through linear regression analysis of the standard curve of the samples. DPPH IC_50_ is the concentration of sample substance that achieves 50% scavenging activity of DPPH free radicals.

### Fe(III) reducing assay

Determination of the Fe(III) reducing activity (FRAP) of sample extract was performed according to the literature (*18*). Three reagents, namely 20 mM FeCl_3_·6H_2_O solution, 10 mM 2,4,6-Tris(2-pyridyl)-*s*-triazine (TPTZ) solution in 40 mM HCl and acetate buffer solution (pH=3.6), were prepared separately. Acetate buffer solution, TPTZ solution, and FeCl_3_·6H_2_O solution were mixed to produce fresh FRAP solution at a volume ratio of 10:1:1. The sample extracts (0.5 mL) were added with 1.95 mL of fresh FRAP solution. The mixture was then incubated for 30 min in the dark before transferred into a 96-well microplate. The absorbance was measured at *λ*=593 nm using an Epoch™ microplate spectrophotometer (Biotek Instrument). The same procedure was repeated for the standard Trolox solution and the calibration curve was obtained as follows:

y=0.0023x+0.1862, R^2^=0.9997 /3/

The results are reported as milligrams of Trolox equivalents per gram extract.

### α-Glucosidase inhibition assay

The α-glucosidase inhibitory activity was measured as described in a previous study with slight modifications (*18*). The positive control was quercetin due to its significant activity. The substrate solution (*p*-NPG) was prepared in 50 mM phosphate buffer (pH=6.5). As for enzyme stock solution, 15 μL of α-glucosidase were added to 4.985 mL of the phosphate buffer and kept in ice-cold water prior use. For the assay, 100 μL of 30 mM buffer solution and 15 μL of α-glucosidase (3 U/mL) together with sample extract were preincubated in 96-well plates at 25 °C for 5 min. Then, 75 μL of *p*-NPG substrate were added and the reaction mixture was incubated for 15 min at 25 °C. The reaction was stopped by adding 50 μL of 2M glycine (pH=10) to the mixture. Blank samples were prepared using the same method as extract samples. However, the enzyme and substrate solution used in the experimental sample were replaced with (50 μL) 30 mM buffer phosphate solution while glycine was replaced with (50 μL) distilled water. The concentrations of sample extracts tested were in the range of 78.13–5000 µg/mL. Absorbance was measured using the Epoch™ microplate spectrophotometer (Biotek Instrument) at *λ*=405 nm. The percentage of α-glucosidase inhibition activity was determined with formula:

Inhibition activity=((Δ*A*_n_–Δ*A*_s_)/Δ*A*_s_)∙100 /4/

where Δ*A*_n_ is the difference between a negative control (with an enzyme) and blank (without an enzyme) and Δ*A*_s_ is the difference in absorption between sample (with enzyme) with blank (without enzyme). Results are expressed as IC_50_ (µg/mL), which implies the concentration of the sample required to achieve 50% enzyme inhibition.

### ^1^H NMR analysis for metabolomics

The ^1^H NMR analysis was done according to the previous methods (*19*, *20*). A mass of 20 mg freeze-dried *C. caesia* extract was put into a 2 mL microcentrifuge tube, and dissolved with a total 0.75 mL of mixture of *V*(methanol-*d*_4_):*V*(KH_2_PO_4_ buffer (pH=6.0) in D_2_O containing 0.1% trimethylsilylpropanoic acid)=1:1. The mixture was vortexed for 1 min and followed by ultrasonication for 15 min at room temperature. Then, the mixture was centrifuged at 17 005×*g* (Hettich Mikro 20, Hettich, Tuttlingen, Germany) for 10 min. After that, a clear supernatant volume of 600 µL was transferred into NMR tubes before subjecting to ^1^H NMR analysis. The ^1^H NMR analysis was performed using a 500 MHz Varian INOVA NMR spectrometer (Varian Inc., Palo Alto, CA, USA), run at a frequency of 499.887 MHz at room temperature. Each ^1^H NMR spectrum contained 64 scans, 20 ppm width, and 3.53 min acquisition time. To enhance the identification of metabolites, 2D NMR *J*-resolved was implemented. The *J*-resolved spectra were analyzed in 50 min and 18 s acquisition time with 8 scans per 128 increments for the axis of the spin-spin coupling constant with spectral widths of 66 Hz and 8 K for the chemical shift axis with spectral widths of 5000 Hz. The relaxation time delay was 1.5 s. Phasing and baseline correction was done using the Chenomx v. 5.1 software (*21*). The spectral were binned into a 0.04 ppm width and regions containing water (*δ*=4.70–5.10 ppm) and residual methanol (*δ*=3.26–3.35 ppm) were excluded from all spectra.

### Statistical analysis

Each analysis was performed with six replicates (*N*=6). Data are expressed as mean±standard deviation. All data analyses were evaluated using one-way and two-way ANOVA with Tukey's comparison in Minitab v. 17 software (*22*). Significant differences between the samples were considered at p<0.05. As for NMR, after binning all the spectra, principal component analysis (PCA) and partial least square (PLS) were performed for multivariate data analysis (MVDA) by SIMCA-P v. 13 software (*23*). The selected scaling method was Pareto to reduce the effect of noise.

## RESULTS AND DISCUSSION

### Effects of drying method and extraction solvent on total phenolic content

Total phenolic content (TPC) of *C. caesia* rhizome and leave extracts acquired from the various groupings of drying methods and *Φ*(ethanol,water) are shown in Table 1. Based on the two-way ANOVA results, drying techniques (p=0.024) and different solvent volume ratios (p=0.000) had a significant effect (p<0.05) on the phenolic content in *C. caesia* rhizome extract. For the leaf extract, the drying factor (p=0.302) did not affect the TPC; instead, the solvent volume ratio (p=0.000) had a significant effect.

**Table 1:** Determination of total phenolic content (TPC) and antioxidant activity (DPPH and FRAP) of black turmeric (*Curcuma caesia*) extracts

Plant part	Drying technique	*Φ*(ethanol,water)	TPC *w*(GAE)/(mg/g)	DPPH IC_50_/(µg/mL)	FRAP *w*(TE)/(mg/g)
Rhizome	FD	100:0	(45.342.1)^a^	(540±58)^gh^	(147.7±8.3)^a^
		80:20	(34.1±3.2)^bc^	(484±64)^h^	(96.8±2.3)^b^
		50:50	(18.4±1.5)^hi^	(1353±167)^c^	(39.1±1.0)^gh^
		0:100	(16.2±1.5)^i^	(2499.64±298)^a^	(29.3±0.7)^ij^
	OD	100:0	(37.3±2.0)^b^	(1376±93)^c^	(55.5±1.6)^d^
		80:20	(29.9±1.0)^de^	(1235±53)^cd^	(55.3±1.2)^de^
		50:50	(22.1±0.5)^g^	(990±32)^de^	(33.2±1.9)^hi^
		0:100	(19.6±0.9)^gh^	(1204±125)^cd^	(26.5±0.9)^jk^
Leaf	FD	100:0	(30.6±1.2)^de^	(1133±33)^cd^	(70.5±3.8)^c^
		80:20	(34.2±0.6)^bc^	(781±27)^efg^	(57.5±2.2)^d^
		50:50	(35.3±1.4)^b^	(661±27)^fgh^	(57.4±1.6)^d^
		0:100	(17.48±0.4)^hi^	(2696±301)^a^	(20.9±1.2)^k^
	OD	100:0	(30.2±2.1)^de^	(1646±18)^b^	(49.4±0.7)^ef^
		80:20	(320±1.1)^cd^	(646±23)^fgh^	(59.0±1.8)^d^
		50:50	(27.6±0.6)^ef^	(864±116)^ef^	(44.2±0.)^fg^
		0:100	(26.26±1.182)^f^	(2694±64)^a^	(44.5±1.2)^fg^
		AA	ND	(25.3±1.4)^i^	ND
		BHA	ND	(44.4±4.5)^i^	ND

Data are expressed as mean value±standard deviation and analyzed using one-way ANOVA. All tests were performed in six replicates (*N*=6). Values with different letters are significantly different (p<0.05). FD=freeze-dried, OD=oven dried, GAE=gallic acid equivalents, TE=Trolox equivalents, AA=ascorbic acid, BHA=butylated hydroxyanisole, ND=no data

The TPC value expressed as GAE of *C. caesia* extracts ranged from (16.2±1.5) to (45.4±2.1) mg/g extract. Rhizome FD extract (*Φ*(ethanol,water)=100:0) revealed significantly (p<0.05) the highest phenolic content compared to the other extracts. For the effect of the drying method, different trends were observed in the samples extracted with different solvents. Freeze-drying was seen to have better ability in the preservation of phenolic compounds than oven drying, on rhizome extract with a higher *Φ*(ethanol,water) ratios which were 100:0 and 80:20. In contrast, OD samples showed higher TPC especially extracts with *Φ*(ethanol,water)=50:50 or 0:100, *i.e.* with water as the solvent. However, the combination of the two factors in this study, FD with extracts with *Φ*(ethanol,water)=100:0 and 80:20 produced significantly higher TPC values than OD combined with extracts with *Φ*(ethanol,water)=50:50 and 0:100, with the highest overall TPC (p<0.05) by rhizome extract of FD *Φ*(ethanol,water)=100:0. These results are in line with literature report which recommends a low-temperature drying technique to retain the active ingredient in plants (*24*). The mechanism of FD in sublimation drying and freezing temperatures (-80 °C) has inhibited the enzyme and microbial activity, thereby preventing biochemical reactions that can alter organoleptic properties, which is associated with molecular structures of the compounds and their concentrations in plant samples (*25*).

Besides the drying effect, the choice of extraction solvent is important regarding the specific nature of the targeted bioactive compounds. According to Do *et al*. (*26*), ethanol is an efficient solvent in extracting polyphenols, and it is recognized as safe for human use. However, water solvents are the opposite due to polyphenol compounds that are more soluble in organic solvents whose polarity is lower than that of water (*27*). Moreover, other studies have also stated that solvents with lower viscosity and density are able to perform better absorption into plant cavities to produce bioactive compounds more easily (*27*, *28*). The density of ethanol (0.789 g/cm^3^) is also lower than that of water (1.0 g/cm^3^), which makes ethanol more effective in the extraction process of phenol compounds (*18*).

The results of this research show that TPC increased with increasing ethanol ratio, except for FD leaf extracts with *Φ*(ethanol,water)=80:20 and 50:50, which contained higher TPC (p<0.05) than extract with ethanol as a solvent (*Φ*=100:0). Meanwhile, aqueous extracts (*Φ*=0:100) from all samples showed lower TPC values (p<0.05) than the extracts with *Φ*(ethanol,water)=100:0 and 80:20. A study by Sajak *et al*. (*29*) on *Ipomoea aquatica* extract also observed higher TPC values in extracts with higher ethanol percentage (50–100%) compared to aqueous extracts. This may be because water solvents have higher potential to extract large macromolecules such as inactive organic acids, proteins and carbohydrates but are less effective in the extracting phenolic compounds that contribute to the antioxidant activity of the extract (*30*). In fact, ethanol is also capable to inactivate enzymes such as polyphenol oxidase that is released into the solvent when plant cell wall breakage occurs during the extraction process (*31*). Thus, more phenolic compounds can be retained in ethanol solvent than in aqueous solvent. Overall, the results of the study indicate that the combination of FD and *Φ*(ethanol,water)=100:0 is a good choice for preservation and extraction of high TPC from *C. caesia*.

### Effect of drying method and extraction solvent on the antioxidant activity

The ability of plant to act as an antioxidant agent is influenced by several factors, which largely depends on the composition of the extract and the analytical test system. The use of only one method cannot describe the antioxidant activity of an extract due to the diversity of molecules and compounds that act as antioxidants and have different mechanisms (*32*). Thus, in the present study two antioxidant assays were conducted; DPPH free radical scavenging and Fe(III) reducing antioxidant power (FRAP) assay. Again, the results of two-way ANOVA showed that the drying technique (p=0.000) and the different solvent ratio (p=0.000) had a significant effect (p<0.001) on the antioxidant activity of *C. caesia* rhizome extract. However, the drying technique (p=0.716) did not have an effect on the DPPH activity of *C. caesia* leaf extract, but was effective in FRAP activity (p=0.001). However, the solvent ratio remained significant (p=0.000) on both antioxidant activities.

The antioxidant activity of *C. caesia* extract is presented in Table 1. DPPH is a stable free radical and is often used in the antioxidant analysis of plant extracts as well as food extracts (*33*). The scavenging activity of DPPH is expressed as IC_50_ (µg/mL), where lower values reflect stronger scavenging activity of the extract. From the results, different trends were observed in the combinations of drying methods and extraction solvents. None of the extracts had lower IC_50_ than the positive control, ascorbic acid ((25.3±1.2) µg/mL) and BHA ((44.4±4.5) µg/mL). This was expected, because among abundance of compounds in the extract, there might have been the compounds that exhibit antagonistic effect with other compounds that possess antioxidant properties. In contrast, the used positive controls were pure active compounds.

In terms of drying method, a similar trend to TPC was observed, in which FD extract obtained using *Φ*(ethanol,water)=100:0 and 80:20 showed better DPPH scavenging activity with lowest IC_50_ values of (539.9±58.0) µg/mL and (484.0±63.8) µg/mL respectively. However, OD rhizome extract showed the opposite trend to TPC, with 50:50 OD extract showing significantly higher scavenging activity than *Φ*=100:0 OD extract. This condition can be explained by the potential presence of other non-phenolic components in the extract that act to accelerate the process of hydrogen transfer to DPPH radicals. Biologically active water-soluble protein from *C. longa*, known as turmerin, has been previously reported with significant antioxidant activity (*34*). Besides, Angel *et al*. (*35*) found that extracted proteins from aqueous *C. caesia* rhizome exhibited high antioxidant activity, comparable to *C. zedoaria*.

Fe(III) reducing power (FRAP), expressed as TE, in *C. caesia* extracts was in the range of 20.9–147.7 mg/g extract. Again, the obtained results show a similar trend to TPC; FD rhizome extracts obtained with higher ethanol ratios showed better Fe(III) reducing activity than OD extract. This finding can be explained by two possibilities: (*i*) the presence of phenolic compounds as major metabolites contributed to the antioxidant activity of the extract, or (*ii*) the effect of applied heat during drying resulted in the decrease of the amount of heat-sensitive metabolites in the OD extract that contribute to the antioxidant activity of *C. caesia* rhizomes. The sublimation process in the FD method leads to the formation of ice crystals in the plant matrix, which in turn creates compressive forces and helps to break the plant cell wall (*36*), so the metabolites can be extracted more easily from the plant matrix.

Overall, extract with only water as the solvent (*Φ*=0:100) showed the lowest FRAP activity and these results were seen to be consistent in all conducted tests. Ethanol in the extraction solvent helps to extract a large number of antioxidant compounds. On the other hand, the low antioxidant activity of the aqueous extract can be explained with the low solubility of antioxidant compounds in aqueous solvents. Sim *et al*. (*37*) also reported that ethanol is more effective for the extraction of polyphenolic compounds than water, influencing the antioxidant, antimicrobial and antityrosinase activities of *Hibiscus cannabinus L*. leaves. Other researchers confirmed that ethanol is more effective in extracting phenolic antioxidants (*38*, *39*). Therefore, it is very crucial to choose the right drying technique and solvent extraction for optimum outcomes.

### Effect of drying method and extraction solvent on α-glucosidase inhibitory activity

The α-glucosidase inhibitory activity is important for the detection of antidiabetic potential of the extract. Based on the previous study, *C. caesia* extract was able to inhibit α-glucosidase activity (*40*). However, information related to the effect of drying techniques and solvent ratios on this bioactivity has not yet been clearly identified. In this study, extracts of rhizomes and leaf of *C. caesia* obtained after drying (p=0.000) and extraction with different solvent ratios (p=0.000) were found to have a significant inhibitory effect (p<0.001) on the activity of α-glucosidase. The results of α-glucosidase inhibition by leaf extract and rhizome of *C. caesia* are shown in Table 2. As can be observed, the extract at the concentration of 625 µg/mL showed inhibitory activity between mild to good with values of 2.96–76.02%. FD rhizome extract had higher inhibitory activity (p<0.05) than OD extract, except for aqueous extract (*Φ*=0:100). Meanwhile, no significant differences (p>0.05) were noticed among the drying techniques and solvents used on the leaf extract except for higher inhibitory activity by FD ethanol (*Φ*=100:0) extract. Again, among all extracts the aqueous one (*Φ*=0:100) had the weakest inhibitory activity (p<0.05).

**Table 2:** Determination of *in vitro* antidiabetic activity of α-glucosidase in black turmeric (*Curcuma caesia*) extracts

Plant part	Drying technique	*Φ*(ethanol,water)		α-glucosidase inhibition/% at *γ*(extract)=625 µg/mL	IC_50_/(µg/mL)
Rhizome	FD	100:0	(76.0±4.8)^a^		(265±39)^de^
		80:20	(62.5±8.8)^b^		(520±49)^cd^
		50:50	(41.1±2.7)^c^		(831±46)^c^
		0:100	(8.6±0.4)^ghi^		ND
	OD	100:0	(28.9±5.5)^d^		(1748±278)^b^
		80:20	(17.6±1.5)^ef^		(2201±94)^b^
		50:50	(13.4±2.0)^efg^		(4498±620)^a^
		0:100	(3.0±0.6)^i^		ND
Leaf	FD	100:0	(19.2±0.6)^e^		ND
		80:20	(10.8±098)^g^		ND
		50:50	(13.7±0.6)^efg^		ND
		0:100	(9.5±1.3)^gh^		ND
	OD	100:0	(8.8±0.6)^ghi^		ND
		80:20	(8.7±0.6)^ghi^		ND
		50:50	(12.6±0.5)^fg^		ND
		0:100	(4.4±0.3)^hi^		ND
		Quercetin	ND		(42.4±3.6)^e^

Data are expressed as mean value±standard deviation and analyzed using one-way ANOVA. All tests were performed in six replicates (*N*=6). Values with different letters are significantly different (p<0.05). FD=freeze-dried, OD=oven dried, ND=no data

The IC_50_ value is the concentration of the extract that can inhibit 50% of α-glucosidase activity, where a lower IC_50_ value means that the extract is more potent. The IC_50_ values of the extracts were determined and compared with quercetin (IC_50_=(42.3±3.6) µg/mL). IC_50_ values were measured only in *Φ*(ethanol,water)=100:0, 80:20 and 50:50 extracts, as the rest of the extracts did not achieve 50% inhibition activity at the observed concentration. The lowest IC_50_ value was shown by the FD rhizome *Φ*=100:0 extract (IC_50_=(265.5±38.6) µg/mL) without significant differences with the rhizome of FD *Φ*=80:20 (IC_50_=(520.0±49.3) µg/mL). This result is probably because of the presence of a large number of polyphenol compounds that have been successfully extracted from the plant matrix. Other studies have also reported the ability of polyphenols in inhibiting the activity of carbohydrate digestive enzymes α-amylase and α-glucosidase (*41*, *42*). The presence of hydroxyl and galloyl groups in the molecular structure of polyphenols contributes to the formation of hydrogen bonds and hydrophobic associations between polyphenols and enzymes, thus assisting in the inhibition of enzyme activity and control of postprandial hyperglycemia in T2DM diabetic patients.

Moreover, previous study by Majumder *et al*. (*40*) also reported the inhibition of α-glucosidase by methanolic extract of *C. caesia* rhizome, but with lower IC_50_ value ((95.4±9.7) μg/mL). This difference might be due to several factors that influenced the extraction and bioactivities of the sample extracts including different geographical origin, preparation process, extraction method, drying technique, the type as well as the concentration of the extraction solvent (*43*). The present study shows that *C. caesia* rhizome was more active compared to the leaf. The combination of FD and higher ethanol solvent ratios of both *Φ*=100:0 and 80:20 in *C. caesia* rhizome extraction exhibited good results throughout the experiment. By considering this outcome, rhizome FD *C. caesia* extracts obtained with different *Φ*(ethanol,water)=100:0, 80:20, 50:50 and 0:100 were selected for further study, using principal component analysis to identify the metabolite differences and find out the correlation between the bioactivities and metabolites using ^1^H NMR-based metabolomics approach.

### Metabolite identification in C. caesia rhizome extracts

**Fig. 1:**
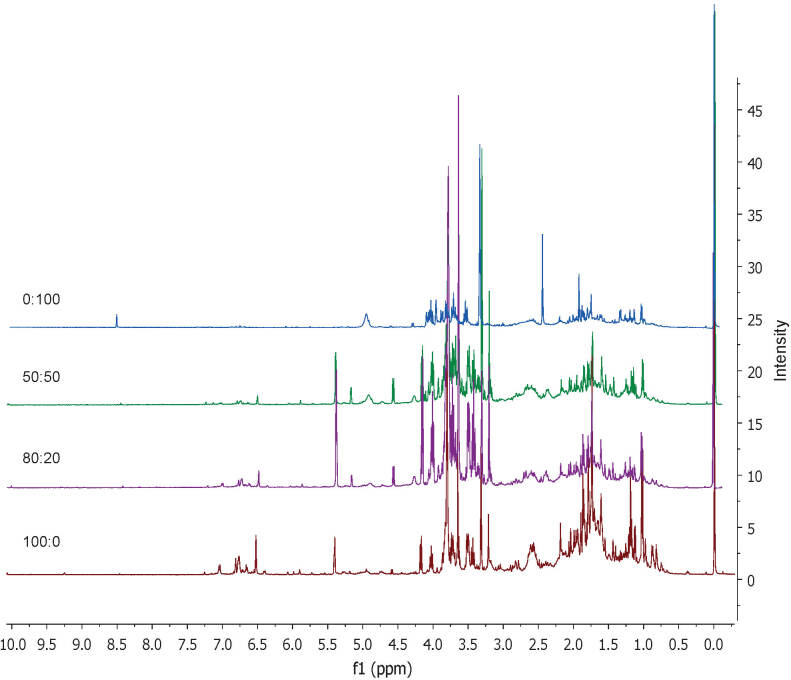
^1^H NMR spectra of *Curcuma caesia* extracts obtained with freeze-drying and *Φ*(ethanol,water)=0:100, 50:50, 80:20 and 100:0 (CD_3_OD/D_2_O, *J*=500 MHz)

**Fig. S2:**
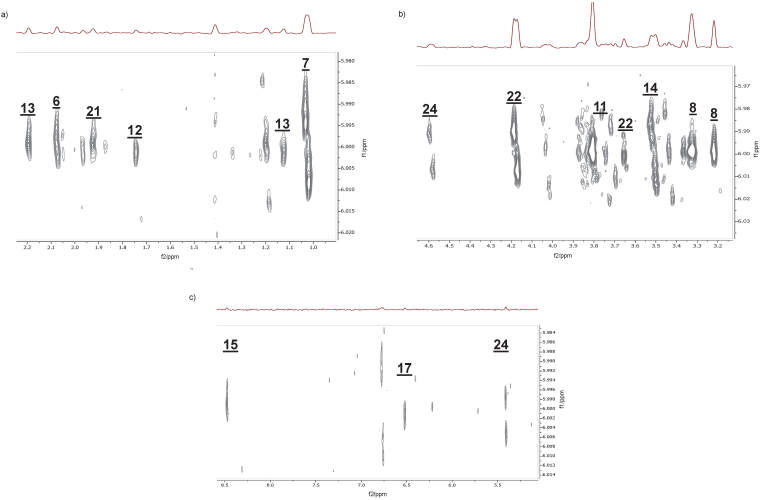
2D *J*-resolved spectra of the freeze-dried (FD) *Curcuma caesia* rhizome extract in the region: a) *δ*=1.0–2.2, b) *δ*=3.2–4.6 and c) *δ*=5.5–8.5 ppm. The observed signals were as follows: 6=β-turmerone, 7=valine, 8=choline, 11=amadannulen, 13=xanthorrhizol derivative, 14=threonine, 15=formic acid, 17=luteolin, 21=selina-4(15),7(11)-dien-8-one, 22=sucrose and 24=β-glucose

The metabolite identification was performed based on spectra from the 1D and 2D NMR (Fig. S2). The ^1^H NMR spectra of FD *C. caesia* rhizome extracts are shown in Fig. 1, which represents the signal of metabolites present in the extract including primary and secondary metabolites. In general, the ^1^H NMR spectra of plant sample extract represents the signals of metabolites that are divided into three regions; namely aliphatic (*δ*=0.5–3.0 ppm), carbohydrate (*δ*=3.0–5.5 ppm) and aromatic (*δ*=5.5–9.0 ppm) region.

A total of 27 metabolites were identified from this plant extract and their characteristic ^1^H NMR signals are shown in Table 3. The identified metabolites were sucrose, glucose, amadannulen, curdione, xanthorrhizol derivative, germacrone, β-turmerone, curcuzederone, 1-hydroxy-1,7-bis(4-hydroxy-3-methoxyphenyl)-(6*E*)-6-heptene-3,4-dione, 1-(4-hydroxy-3,5-dimethoxyphenyl)-7-(4-hydroxy-3-methoxyphenyl)-(l*E*,6*E*)-1,6-heptadiene3,4-dione, 13-hydroxygermacrone, xanthorrhizol, luteolin, threonine, valine, choline, formic acid, selina-4(15),7(11)-dien-8-one, zedoardiol, zedoalactone A, zedoalactone B, curzerene, zerumin B, gallic acid, demethoxycurcumin and curcumin. The detection of these metabolites was done by comparing the NMR chemical shifts and coupling constants with other research findings as well as 2D NMR experiments. The signal differences, especially in the carbohydrate and aromatic regions, were seen in all extracts. However, the ^1^H NMR signals in these regions were less prominent in *Φ*=0:100 extract than in other extracts. Therefore, more attention was given to these regions, as they are likely to act as a differentiating factor between the extracts.

**Table 3:** Identification of ^1^H NMR metabolites in freeze-dried rhizome extract of *Curcuma caesia*

No. ID	Metabolite	^1^H NMR signal *δ*/ppm	*Φ*(ethanol,water)			
0:100	50:50	80:20	100:0
1	Curcumin	*δ*_s_=3.89, 3.90, 7.26	-	+	+	+
2	Demethoxycurcumin	*δ*_s_=3.94, 3.95, 5.90	+	+	+	+
3	1-Hydroxy-1,7-bis(4-hydroxy-3-methoxyphenyl)-(6*E*)-6-heptene-3,4-dione	*δ*_d_=7.04 (*J*=7.85 Hz)	-	-	+	+
4	1-(4-Hydroxy-3,5-dimethoxyphenyl)-7-(4-hydroxy-3-methoxyphenyl)-(l*E*,6*E*)-1,6-heptadiene-3,4-dione	*δ*_s_=6.81	-	+	+	+
5	13-Hydroxygermacrone	*δ*s=1.44, 1.61	+	+	+	+
6	β-turmerone	*δ*_s_=1.80, 2.05, 2.07	+	+	+	+
7	Valine	*δ*_d_=1.03 (*J*=6.95 Hz), *δ*_d_=1.13 (*J*=6.05 Hz)	+	+	+	+
8	Choline	*δ*_s_=3.22, 3.3	+	-	+	+
9	Curdione	*δ*_d_=0.88 (*J*=7.55 Hz)	-	-	-	+
10	Curcuzederone	*δ*_s_=1.16, 1.34, 3.76	+	+	+	+
11	Amadannulen	*δ*_s_=0.85, 3.80, 3.81	+	+	-	+
12	Germacrone	*δ*_s_=1.61, 1.74s	-	-	-	+
13	Xanthorrhizol derivative	*δ*_s_=1.10, 1.15, 2.19 *δ*_d_=6.66 (*J*=7.65 Hz), *δ*_d_=6.66 (*J*=7.75 Hz)	+	+	+	+
14	Threonine	*δ*_d_=1.33 (*J*=6.75 Hz), *δ*_d_=3.50 (*J*=4.0 Hz)	+	+	+	+
15	Formic acid	*δ*_s_=8.47	+	+	+	+
16	Gallic acid	*δ*_s_=7.15	+	+	+	+
17	Luteolin	*δ*_s_=6.52	+	+	+	+
18	Zedoardiol	*δ*_s_=1.41, 1.71, 1.98	-	-	+	+
19	Zedoalactone A	*δ*_s_=1.56	+	+	+	+
20	Zedoalactone B	*δ*_s_=1.75, 1.90, 6.07	+	+	+	+
21	Selina-4(15),7(11)-dien-8-one	*δ*_s_=1.68, 1.95, 1.97	+	+	+	+
22	Sucrose	*δ*_s_=3.65, *δ*_d_=4.18 (*J*=8.7 Hz), *δ*_d_=5.41 (*J*=3.65 Hz	+	+	+	+
23	α-glucose	*δ*_t_=3.43 (*J*=9.7, 9.4 Hz), *δ*_t_=3.44 (*J*=9.5, 9.45 Hz), *δ*_d_=5.19 (*J*=3.7 Hz)	+	+	+	+
24	β-glucose	*δ*_d_=4.59 (*J*=7.9 Hz)	+	+	+	+
25	Zerumin B	*δ*_s_=0.68, 2.44	+	+	-	+
26	Curzerene	*δ*_s_=1.81	-	+	+	-
27	Xanthorrhizol	*δ*_s_=1.87, 1.88, 2.00	+	+	+	+

Metabolites of various classes were indicated by the ^1^H NMR signals, including amino acids, organic acids, phenolic, carbohydrates, and terpenoids. Amino acids such as threonine (*δ*_d_=1.33 ppm, *J*=6.75 Hz, *δ*_d_=3.50 ppm, *J*=4.0 Hz) and valine (*δ*_d_=1.03 ppm, *J*=6.95 Hz, *δ*_d_=1.13 ppm, *J*=6.05 Hz) were successfully identified (*44*, *45*). The phenolic compounds detected in aromatic region were curcumin, 1-(4-hydroxy-3,5-dimethoxyphenyl)-7-(4-hydroxy-3-methoxyphenyl)-(l*E*,6*E*)-1,6-heptadiene3,4-dione and demethoxycurcumin, 1-hydroxy-1,7-bis(4-hydroxy-3-methoxyphenyl)-(6*E*)-6-heptene-3,4-dione. Besides, in the same region there were also signals for zedoalactone B, luteolin, xanthorrhizol derivative, gallic acid and formic acid (*δ*_s_=6.07 ppm, *δ*_s_=6.52ppm, *δ*_d_=6.66 (J=7.65Hz), *δ*_s_=7.15 ppm, *δ*_s_=8.47 ppm) (*14*, *29*). Furthermore, metabolites consisting of sugars such as sucrose, α-glucose and β-glucose were identified based on signals from the carbohydrate region (*δ*=3.0-5.5 ppm) (*46*).

Other than that, metabolites from the terpene group most commonly found as constituents in essential oils were also present in the extracts. Zerumin B was among those detected with two singlet chemical shifts *δ*_s_=0.68 ppm and *δ*_s_=2.44 ppm. Sesquiterpenes such as curcuzederone, curdione, curzerene, germacrone and selina-4(15),7(11)-dien-8-one were successfully found. Two singlets at *δ*_s_=1.44 ppm and *δ*_s_=1.61 ppm were assigned to 13-hydroxygermacrone. Signals at *δ*_s_=1.88 ppm and *δ*_s_=2.00 ppm were matched with xanthorrhizol, while the three singlet signals *δ*_s_=1.10 ppm, *δ*_s_=1.15 ppm, *δ*_s_=2.19 ppm, and one doublet (*δ*_d_=6.66 ppm, J=7.65Hz) referred to the xanthorrhizol derivative. Choline was also identified at two chemical shifts *δ*_s_=3.22 ppm and *δ*_s_=3.32 ppm.

### Principal component analysis of C. caesia rhizome extracts

**Fig. 2:**
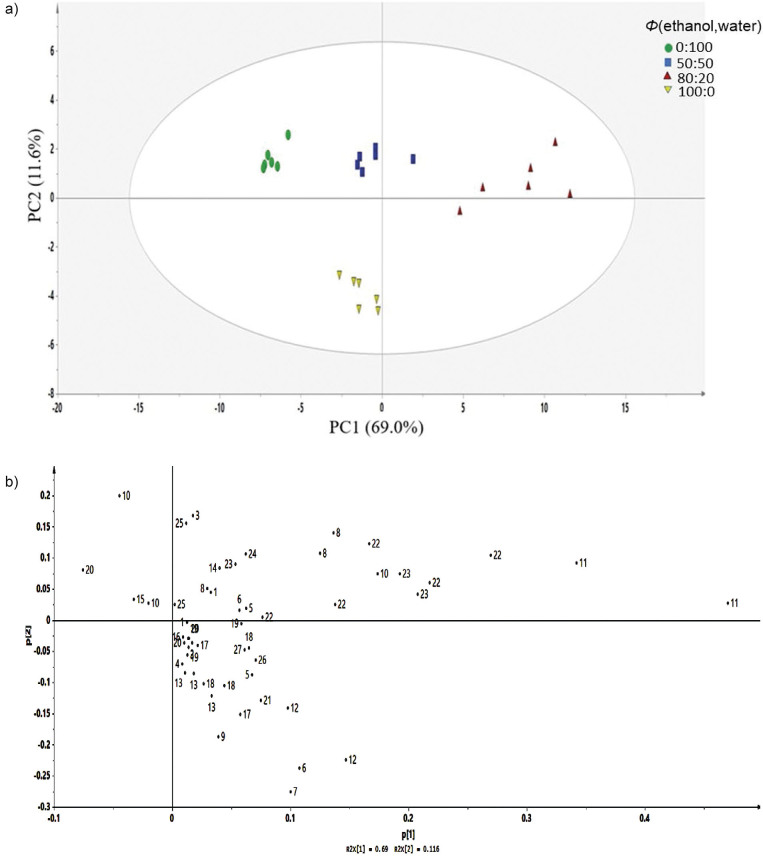
Score plot (a) and loading plot (b) of freeze-dried (FD) rhizome of *Curcuma caesia* extract. 1=curcumin, 2=demethoxycurcumin, 3=1-hydroxy-1,7-bis(4-hydroxy-3-methoxyphenyl)-(6*E*)-6-heptene-3,4-dione, 4=1-(4-hydroxy-3,5-dimethoxyphenyl)-7-(4-hydroxy-3-methoxyphenyl)-(l*E*,6*E*)-1,6-heptadiene-3,4-dione, 5=13-hydroxygermacrone, 6=β-turmerone, 7=valin, 8=choline, 9=curdione, 10=curcuzederone, 11=amadannulen, 12=germacrone, 13=xanthorrhizol derivative, 14=threonine, 15=formic acid, 16=gallic acid, 17=luteolin, 18=zedoardiol, 19=zedoalactone A, 20=zedoalactone B, 21=selina-4(15),7(11)-dien-8-one, 22=sucrose, 23=α-glucose, 24=β-glucose, 25=zerumin B, 26=curzerene and 27=xanthorrhizol

The comparison of the metabolite profile of *C. caesia* extracts obtained with different *Φ*(ethanol,water) was done using the MVDA method as the principal component analysis (PCA) model. This method helps to identify differences or similarities between samples with the help of score plots. Fig. 2a shows a clear separation between extracts in the PCA score plot without any significant outliers. The total variance of the model was 80.6%, for which PC1 contributed by 69.0% and PC2 with 11.6%. This model is considered a good model because its goodness of fit, R2X (cum) and Q2(cum)>0.5, as well as the difference between these two, was <0.3, showing the uniformity of each extract in contributing to the separation of each group (*29*).

As shown in Fig. 2a, four clusters can be seen representing each extract, *Φ*(ethanol,water)=0:100, 50:50, 80:20 and 100:0. Based on the score plot, the *Φ*=100:0 extract was separated from the others by PC2, meanwhile the *Φ*=80:20 extract was separated from other samples by PC1. Through the score plot, separate samples form clusters based on differences in ethanol and water solvent ratios, indicating the possibility of metabolic changes due to the solvent ratios used. Meanwhile, the loading plot (Fig. 2b) completes the PCA model by showing phytochemical metabolites that contribute to cluster separation between extracts. The identification of metabolites allows compounds that contribute to the separation between study samples and compounds commonly found in samples to be identified.

**Fig. 3:**
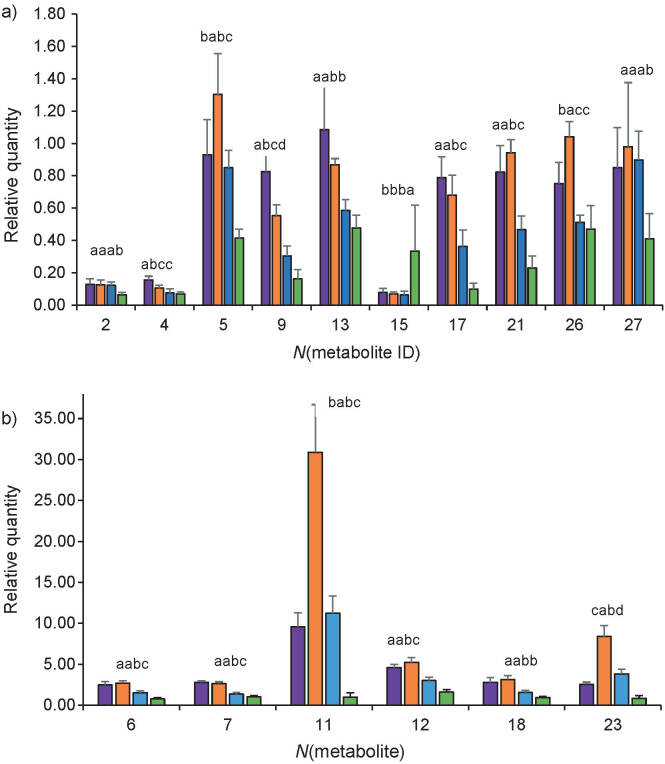
Relative quantification of freeze-dried (FD) *Curcuma caesia* rhizome metabolites extracted with *Φ*(ethanol,water)=0:100 (green), 50:50 (blue), 80:20 (orange) and 100:0 (purple). Letters show significant differences (p<0.05): a) 2=demethoxycurcumin, 4=1-(4-hydroxy-3,5-dimethoxyphenyl)-7-(4-hydroxy-3-methoxyphenyl)-(l*E*,6*E*)-1,6-heptadiene-3,4-dione, 5=13-hydroxygermacrone, 9=curdione, 13=xanthorrhizol derivative, 15=formic acid, 17=luteolin, 21=selina-4(15),7(11)-dien-8-one, 26=curzerene and 27=xanthorrhizol, and b) 6=β-turmerone, 7=valin, 11=amadannulen, 12=germacrone, 18=zedoardiol, 23=α-glucose

According to the loading plot (Fig. 2b), most of the metabolites were concentrated in the central and right quadrant, indicating higher metabolites constituents in extracts with more ethanol ratio (*Φ*=100:0 and 80:20). However, 13-hydroxygermacrone (5), amadannulen (11), α-glucose (23), and curzerene (26) were seen as the compounds that separate *Φ*=80:20 extract from other extracts (*Φ*=0:100, 50:50 and 100:0). Based on the relative quantification of the metabolites in the extracts (Fig. 3), metabolite phenolic 1-(4-hydroxy-3,5-dimethoxyphenyl)-7-(4-hydroxy-3-methoxyphenyl)-(l*E*,6*E*)-1,6-heptadiene3,4-dione (4) and sesquiterpene curdione (9) were highest in *Φ*=100:0 extract. These are the compounds suspected to be responsible for the higher antioxidant and antidiabetic activities in the *Φ*=100:0 extracts compared to others. Interestingly, the demethoxycurcumin (2), β-turmerone (6), valine (7), germacrone (12), xanthorrhizol derivative (13), luteolin (17), zedoardiol (18), selina-4 (15), 7(11)-dien-8-one (21) and xanthorrhizol (27) were found highest in both *Φ*=100:0 and 80:20 extracts. The result showed both of the extracts contained almost similar bioactive compounds with various concentrations, hence less ’discriminating power’ that highlights the compound properties of the extract. The result can be due to the extraction solvent greatly affecting the quantity of the extracted compounds. Difference in the distribution of the compounds could be attributed to the nature of the metabolites and their solubility in the extraction solvents.

### Correlation between in vitro bioassay and metabolites

Correlation was analysed with the help of PLS as an MVDA-supervised multivariate model. The PLS helps in determining the relationship between bioactivities of the sample with the phytochemicals that it contains. In this study, PLS was used to determine the correlation between bioactivities (antioxidants and α-glucosidase inhibitory activity) of *C. caesia* extract with the identified metabolites. PLS separates the data into two blocks, namely block X (predictor variable: ^1^H NMR metabolite signal) and block Y (reaction variable: bioactivity). Results that are expressed in IC_50_ values give clearer information in determining the effectiveness of the extracts. However, the IC_50_ values obtained in this study could not be used due to insufficient data by one of the samples, which was at the unidentifiable reading range. Therefore, bioactivity data of the extracts used were the inhibitory percentage (%) for DPPH and α-glucosidase assays, while mg Trolox equivalents per g extract were used for FRAP.

**Fig. S3:**
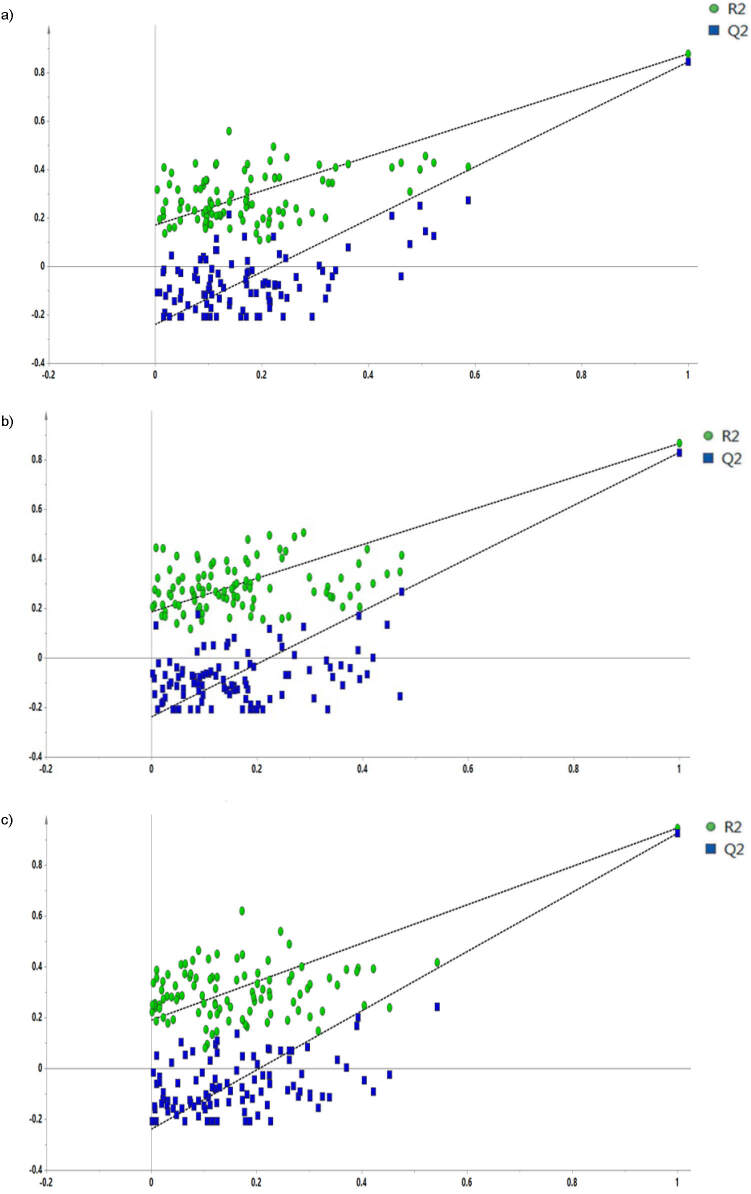
Validation by the permutation test of bioassay: a) DPPH scavenging activity, b) α-glucosidase inhibitory activity and c) FRAP using 100 permutations for the partial least square (PLS) model. R2=measure of model fit to the original data, Q2=internal measure of consistency between the original and cross-validation predicted data

The PLS biplot is the result of the combination of score plot and loading plot. The model validation was executed by permutation test with 100 permutations, to evaluate its goodness of fit and predictive power. It was also validated by the evaluation of the regression of the observed and predicted values (*47*). The comparison of the R2 and Q2 of the original model with those where the Y variable has been permuted randomly provides an unbiased validity and degree of overfitting estimation of the PLS model (*48*). In this study, all PLS models showed the validity with R2Y intercept <0.3–0.4 and Q2Y intercept <0.05 (Fig. S3), suggesting that the models fulfilled the criteria of excellent validation and they were safe from over-fitting. The CV ANOVA results also revealed that all models were significant (p<0.05).

**Fig. 4:**
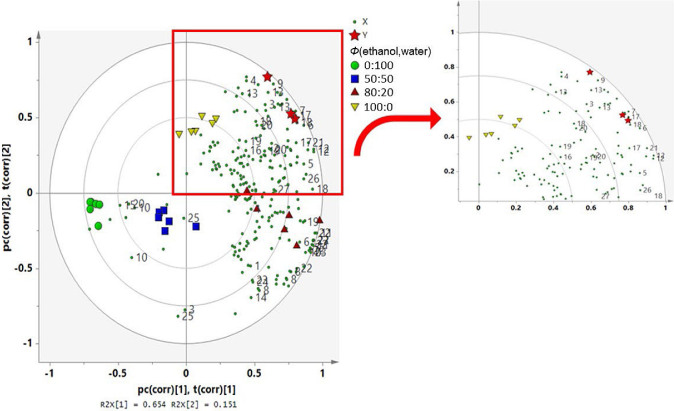
Partial least square (PLS) bi-plots showing correlations between the freeze-dried (FD) *Curcuma caesia* rhizome extracts obtained with *Φ*(ethanol,water)=0:100, 50:50, 80:20 and 100:0, and bioactivity (see Table 3 for metabolite identification). X=predictor variable, ^1^H NMR metabolite signal, Y=reaction variable, bioactivity

From the results in Fig. 4, it can be seen that the PLS biplot had the same pattern as PCA, whereby four clusters were clearly observed separating the extracts. The ethanolic extract (*Φ*=100:0) cluster, which was located in the upper right quadrant of the plot, was seen separated from the other extract clusters and projected close to the bioactivities (α-glucosidase, DPPH, FRAP), thus suggesting strong correlation between them. The position of the *Φ*=80:20 extract cluster was also close to the antioxidant activity of DPPH and FRAP, suggesting their strong activities in the bioassay tests compared to the *Φ*=50:50 and 0:100 extracts located in the lower-left quadrant of the plot. The position of *Φ*=50:50 and 0:100 extracts was far from bioactivities, indicating a very low correlation with the reaction variables. This finding is in accordance with the bioassay results where the *Φ*=100:0 extract had the highest antioxidant and antidiabetic activities, while *Φ*=0:100 extract had the lowest activities in the conducted DPPH, FRAP and α-glucosidase tests. As for *Φ*=80:20 extract, although it is located in the same right region quadrant and close to the antioxidant activities, it had no significant difference (p>0.05) with *Φ*=100:0 extract in DPPH scavenging activity. This indicates that these two extracts have similar levels of efficiency of the extraction of metabolite compounds actively involved in the radical scavenging activity of DPPH. However, for Fe(III) reducing activity (FRAP) and α-glucosidase inhibition, it was found that *Φ*=100:0 extract gave higher activity (p<0.05) than *Φ*=80:20, 50:50 and 0:100 extracts. These results are also supported by previous studies that reported that 100% ethanol extracts had better biological activities and contained more metabolites than aqueous extracts (*29*, *47*, *49*).

Metabolites that contributed greatly to the bioactivities of the extract in PLS model were further evaluated through their variable important in projections (VIP) value, which separates the extract clusters with higher metabolites and bioactivity. VIP values higher than 0.7 are usually suggested as the key signals that affected PLS model projection (*20*). According to the PLS biplot, several metabolites were found to be responsible for the antioxidant and antidiabetic activities of the *C. caesia* extracts. They were xanthorrhizol derivative (VIP=6.40), zedoalactone B (VIP=4.86), xanthorrhizol (VIP=3.68), selina-4(15),7(11)-dien-8-one (VIP=3.32), valine (VIP=3.12), zedoalactone A (VIP=2.63), 13-hydroxygermacrone (VIP=2.14), curdione (VIP=1.99), zedoardiol (VIP=1.95), luteolin (VIP=1.76), demethoxycurcumin (VIP=1.75), 1-hydroxy-1,7-bis (4-hydroxy-3-methoxyphenyl)-(6*E*)-6-heptene-3,4-dione (VIP=1.24), β-turmerone (VIP=1.05) and 1-(4-hydroxy-3,5-dimethoxyphenyl)-7-(4-hydroxy-3-methoxyphenyl)-(l*E*,6*E*)-1,6-heptadiene-3,4-dione VIP=0.73). All these metabolites had greater impact on the PLS model compared to other metabolites. Overall, high phenolic content in *Φ*=100:0 extract may be attributed to the examined biological activities. This is also supported by other findings confirming that antioxidant and antidiabetic activities of plant extracts are mainly due to their phenolic content (*50*, *51*). Moreover, xanthorrhizol (*52*), luteolin (*53*) and demethoxycurcumin (*54*) has been reported previously to possess antioxidant and antidiabetic activities.

Based on the overall results, it can be suggested that both *Φ*=100:0 and 80:20 extracts have the same antioxidant potential, with almost similar metabolite content. Complete metabolomic identification of the extracts may increase the possibility to differentiate between the extracts. However, it is impossible to identify the whole metabolome of the crude extract due to the abundance of metabolites in it. The phenolic metabolites 1-(4-hydroxy-3,5-dimethoxyphenyl)-7-(4-hydroxy-3-methoxyphenyl)-(l*E*,6*E*)-1,6-heptadiene-3,4-dione (4) and sesquiterpene curdione (9), which showed strong correlation with α-glucosidase activity (Fig. 3), were found to have the significantly (p<0.05) highest content in ethanolic extract (*Φ*=100:0). This is also supported by the observed *in vitro* test results, where the ethanolic extract exhibited the lowest IC_50_ values value compared to the other extracts (Table 2). Thus, ethanolic *C. caesia* extract can be suggested as a potential sample with the highest effectiveness in inhibiting α-glucosidase activity.

## CONCLUSIONS

In conclusion, the present study demonstrates that *Curcuma caesia* leaves and rhizome that were dried (by freeze- and oven drying method) and extracted with different ethanol and water volume ratios (*Φ*(ethanol,water)=100:0, 80:20, 50:50 and 0:100) had variations in their total phenolic content (TPC), DPPH radical scavenging, FRAP and α-glucosidase inhibition activity. Based on the *in vitro* tests, freeze-dried ethanolic *C. caesia* rhizome extract presented higher TPC, FRAP activity and α-glucosidase inhibition capacity than the other extracts. In DPPH assay, freeze-dried (FD) *Φ*=100:0 and 80:20 *C. caesia* rhizome extracts exhibited better results than the other extracts, with no significant difference between the two. From the bioassay data, it was concluded that FD *C. caesia* rhizome extracts have higher TPC and better antioxidant and antidiabetic activities. Therefore, the FD rhizome extracts were further evaluated with ^1^H NMR metabolomics for profiling and identification of metabolites responsible for variation between the extracts. The most active (FD *Φ*=100:0) and the second most active (FD *Φ*=80:20) extracts in antioxidant activity contained a large amount of phenolic and sesquiterpenes compounds, however, FD *Φ*=100:0 extract showed better α-glucosidase inhibition activity with higher content of metabolites 1-(4-hydroxy-3,5-dimethoxyphenyl)-7-(4-hydroxy-3-methoxyphenyl)-(l*E*,*6E*)-1,6-heptadiene-3,4-dione and curdione. Despite the valuable information provided in this study, further investigation is required to assess their safety and long-term pharmaceutical benefits in the *in vivo* model, to provide a guide for the development of pharmacological products or functional foods.

## References

[1] Paliwal P Pancholi SS Patel RK. Pharmacognostic parameters for evaluation of the rhizomes of *Curcuma caesia.* J Adv Pharm Technol Res. 2011;2(1):56–61. 10.4103/2231-4040.79811 22171294 PMC3217681

[2] Saikia B Borthakur SK. Use of medicinal plants in animal healthcare - A case study from Gohpur, Assam. Indian J Tradit Knowl. 2010;9(1):49–51.

[3] Ghosh A Parthadeb G Chatterjee P. Evaluation of antimicrobial and anti-fungal potential of (Z)-7-methoxy-1, 5-dihydrobenzo[c]oxepine, isolated from *Curcuma caesia* Roxb. J Sci Innov Res. 2013;2(4):745–50.

[4] Das S Bordoloi PKR Phukan D Singh SR. Study of the anti-ulcerogenic of ethanolic extract of rhizome of *Curcuma caesia* (EECC) against gastic ulcers in experimental animals. Asian J Pharm Clin Res. 2012;5:200–3.

[5] Bharathi SV Anuradha V Ahmad ALI Tajo SM. Thrombolytic activity of *Curcuma amada* and *Curcuma caesia.* Asian J Pharm Clin Res. 2017;10(2):317–8. 10.22159/ajpcr.2017.v10i2.15694

[6] Sawant SB Bihani G Mohod S Bodhankar S. Evaluation of analgesic and anti-inflammatory activity of methanolic extract of *Curcuma caesia* Roxb. rhizomes in laboratory animals. Int J Pharm Pharm Sci. 2014;6(2):243–7.

[7] Karmakar I Dolai N Saha P Sarkar N Bala A Ki HP. Scavenging activity of *Curcuma caesia* rhizome against reactive oxygen and nitrogen species. Orient Pharm Exp Med. 2011;11:221–8. 10.1007/s13596-011-0030-6

[8] Pakkirisamy M Kalakandann SK Ravichandran K. Phytochemical screening, GC-MS, FT-IR analysis of methanolic extract of *Curcuma caesia* Roxb (Black Turmeric). Pharmacogn. 2017;9(6):952–6. 10.5530/pj.2017.6.149

[9] Chaturvedi M Rani R Sharma D Yadav JP. Effect of temperature and pressure on antimycobacterial activity of *Curcuma caesia* extract by supercritical fluid extraction method. Int J Mycobacteriol. 2020;9(3):296–302. 10.4103/ijmy.ijmy_113_20 32862164

[10] Sarangthem K Haokip MJ. Bioactive components in *Curcuma caesia* Roxb. grown in Manipur. Int J Life Sci (Kathmandu). 2010;5:113–5.

[11] Sethi S Brietzke E. Omics-based biomarkers: Application of metabolomics in neuropsychiatric disorders. Int J Neuropsychopharmacol. 2015;19(3):pyv096. 10.1093/ijnp/pyv096 26453695 PMC4815467

[12] Alonso A Marsal S Julià A. Analytical methods in untargeted metabolomics: State of the art in 2015. Front Bioeng Biotechnol. 2015;3:23. 10.3389/fbioe.2015.00023 25798438 PMC4350445

[13] Markley JL Brüschweiler R Edison AS Eghbalnia HR Powers R Raftery D The future of NMR-based metabolomics. Curr Opin Biotechnol. 2017;43:34–40. 10.1016/j.copbio.2016.08.001 27580257 PMC5305426

[14] Mediani A Abas F Khatib A Maulidiani H Shaari K Choi YH ^1^H-NMR-based metabolomics approach to understanding the drying effects on the phytochemicals in *Cosmos caudatus.* Food Res Int. 2012;49(2):763–70. 10.1016/j.foodres.2012.09.022

[15] Mediani A Abas F Khatib A Tan CP. *Cosmos caudatus* as a potential source of polyphenolic compounds: Optimisation of oven drying conditions and characterisation of its functional properties. Molecules. 2013;18(9):10452–64. 10.3390/molecules180910452 23994970 PMC6270166

[16] Reenu J Azeez S Bhageerathy C. *In vitro* antioxidant potential in sequential extracts of *Curcuma caesia* Roxb. Rhizomes. Indian J Pharm Sci. 2015;77(1):41–8. 10.4103/0250-474X.151596 25767317 PMC4355881

[17] Azmir J Zaidul ISM Rahman MM Sharif KM Mohamed A Sahena F Techniques for extraction of bioactive compounds from plant materials: A review. J Food Eng. 2013;117(4):426–36. 10.1016/j.jfoodeng.2013.01.014

[18] Zahwal EN Chen PF Ibrahim NNA Razali NSM Abdul Rahman H. Effect of different drying methods and solvents on phenolic content, antioxidant and anti-hyperglycemic activities of *Melastoma malabathricum* leaves extract. Malays Appl Biol. 2018;47(5):47–53.

[19] Kim HK Choi YH Verpoorte R. NMR-based metabolomic analysis of plants. Nat Protoc. 2010;5:536–49. 10.1038/nprot.2009.237 20203669

[20] Kim HK Choi YH Verpoorte R. NMR-based plant metabolomics: Where do we stand, where do we go? Trends Biotechnol. 2011;29(6):267–75. 10.1016/j.tibtech.2011.02.001 21435731

[21] Chenomx, v. 5.1, Chenomx Inc., Edmonton, AB, Canada; 2010. Available from: https://www.chenomx.com/products/.

[22] Minitab, v. 17, Minitab Ltd, Coventry, UK; 2014. Available from: https://www.minitab.com/en-us/products/minitab/.

[23] SIMCA-P. v. 13, Sartorius AG, Goettingen, Germany; 2012. Available from: https://www.sartorius.com/en/products/process-analytical-technology/data-analytics-software/mvda-software/simca.

[24] Aboltins A, Kic P. Research in some medical plant drying process. In: Proceedings of the international conference Engineering for Rural Development; 2016 March 25-27, Jeglava, Latvia: Latvia University of Agriculture; 2016. pp. 1145–50.

[25] Rahimmalek M Goli SAH. Evaluation of six drying treatments with respect to essential oil yield, composition and color characteristics of *Thymys daenensis* subsp. *daenensis* Celak leaves. Ind Crops Prod. 2013;42(1):613–9. 10.1016/j.indcrop.2012.06.012

[26] Do QD Angkawijaya AE Tran-Nguyen PL Lien HH Soetaredjo FE Ismadji S Effect of extraction solvent on total phenol content, total flavonoid content, and antioxidant activity of *Limnophila aromatica.* J Food Drug Anal. 2014;22(3):296–2. 10.1016/j.jfda.2013.11.001 28911418 PMC9354875

[27] Özbek HN Halahlih F Göğüş F Koçak Yanık D Azaizeh H. Pistachio (*Pistacia vera* L.) hull as a potential source of phenolic compounds: Evaluation of ethanol–water binary solvent extraction on antioxidant activity and phenolic content of pistachio hull extracts. Waste Biomass Valoriz. 2020;11:2101–10. 10.1007/s12649-018-0512-6

[28] Zuorro A Iannone A Lavecchia R. Water–organic solvent extraction of phenolic antioxidants from brewers’ spent grain. Processes (Basel). 2019;7(3):126. 10.3390/pr7030126

[29] Sajak AAB Abas F Ismail A Khatib A. Effect of different drying treatments and solvent ratios on phytochemical constituents of *Ipomoea aquatica* and correlation with α-glucosidase inhibitory activity. Int J Food Prop. 2016;19:2817–31. 10.1080/10942912.2016.1141295

[30] Jara-Palacios MJ Gon Ã. §alves S, Heredia FJ, Hernanz D, Romano A. Extraction of antioxidants from winemaking byproducts: Effect of the solvent on phenolic composition, antioxidant and anti-cholinesterase activities, and electrochemical behaviour. Antioxidants. 2020;9(8):675. 10.3390/antiox9080675 32731540 PMC7465776

[31] Sripum C Kukreja RK Charoenkiatkul S Kriengsinyos W Suttisansanee U. The effect of extraction conditions on antioxidant activities and total phenolic contents of different processed Thai Jasmine rice. Int Food Res J. 2017;24(4):1644–50.

[32] Ioannou I, Chaaban H, Slimane M, Ghoul M. Origin of the variability of the antioxidant activity determination of food material. In: Ekinci D, editor. Biotechnology. London, UK: InTech; 2015. pp. 77–9. 10.5772/60453 10.5772/60453

[33] Dewi YSK Simamora CJK Fadly D. Antioxidant and antimicrobial activities of methanolic extracts of *Scorodocarpus borneensis* Becc. Syst Rev Pharm. 2020;11(7):246–52. 10.31838/srp.2020.7.39

[34] Lekshmi PC Arimboor R Raghu KG Menon AN. Turmerin, the antioxidant protein from turmeric (*Curcuma longa*) exhibits antihyperglycaemic effects. Nat Prod Res. 2012;26(17):1654–8. 10.1080/14786419.2011.589386 21972920

[35] Angel GR Vimala B Nambisan B. Antioxidant and anti-inflammatory activities of proteins isolated from eight *Curcuma* species. Phytopharmacol. 2013;4(1):96–1.

[36] Oyinloye TM Yoon WB. Effect of freeze-drying on quality and grinding process of food produce: A review. Processes (Basel). 2020;8(3):354–77. 10.3390/pr8030354

[37] Sim YY Ong WTS Nyam KL. Effect of various solvents on the pulsed ultrasonic assisted extraction of phenolic compounds from *Hibiscus cannabinus* L. leaves. Ind Crops Prod. 2019;140:111708. 10.1016/j.indcrop.2019.111708

[38] Maulana TI Falah S Andrianto D. Total phenolic content, total flavonoid content, and antioxidant activity of water and ethanol extract from Surian (*Toona sinensis*) leaves. IOP Conf Ser Earth Environ Sci. 2019;299:012021. 10.1088/1755-1315/299/1/012021

[39] Nguyen V Van Vuong Q Bowyer M Van Altena I Scarlett C. Effects of different drying methods on bioactive compound yield and antioxidant capacity of *Phyllanthus amarus.* Dry Technol. 2015;33(8):1006–17. 10.1080/07373937.2015.1013197

[40] Majumder P. Mazumder *S*, Chakraborty M, Chowdhury SG, Karmakar S, Haldar PK. Preclinical evaluation of Kali Haldi (*Curcuma caesia*): A promising herb to treat type-2 diabetes. Orient Pharm Exp Med. 2017;17:161–9. 10.1007/s13596-017-0259-9

[41] Asgar AMD. Anti-diabetic potential of phenolic compounds: A review. Int J Food Prop. 2013;16(1):91–103. 10.1080/10942912.2011.595864

[42] Lin D Xiao M Zhao J Li Z Xing B Li X An overview of plant phenolic compounds and their importance in human nutrition and management of type 2 diabetes. Molecules. 2016;21(10):1374. 10.3390/molecules21101374 27754463 PMC6274266

[43] Azwanida NN. A review on the extraction methods use in medicinal plants, principle, strength and limitation. Med Aromat Plants. 2015;4(3):1000196. 10.4172/2167-0412.1000196

[44] Rahman HA Sahib NG Saari N Abas F Ismail A Mumtaz MW Anti-obesity effect of ethanolic extract from *Cosmos caudatus* Kunth leaf in lean rats fed a high fat diet. BMC Complement Altern Med. 2017;17(1):122–39. 10.1186/s12906-017-1640-4 28228098 PMC5322639

[45] Che Zain MS Lee SY Mad Nasir N Fakurazi S Shaari K. Metabolite characterization and correlations with antioxidant and wound healing properties of oil palm (*Elaeis guineensis* Jacq.) leaflets *via* ^1^H-NMR-based metabolomics approach. Molecules. 2020;25(23):5636. 10.3390/molecules25235636 33265992 PMC7731087

[46] Pramai P Abdul Hamid NA Mediani A Maulidiani M Abas F Jiamyangyuen S. Metabolite profiling, antioxidant, and α-glucosidase inhibitory activities of germinated rice: Nuclear-magnetic-resonance-based metabolomics study. J Food Drug Anal. 2018;26(1):47–57. 10.1016/j.jfda.2016.11.023 29389588 PMC9332653

[47] Awin T Mediani A Maulidiani H Shaari K Mohd Faudzi SM Sukari MAH Phytochemical profiles and biological activities of Curcuma species subjected to different drying methods and solvent systems: NMR-based metabolomics approach. Ind Crops Prod. 2016;94:342–52. 10.1016/j.indcrop.2016.08.020

[48] Lee SY Mediani A Maulidiani M Khatib A Ismail IS Zawawi N Comparison of partial least squares and random forests for evaluating relationship between phenolics and bioactivities of *Neptunia oleracea.* J Sci Food Agric. 2018;98(1):240–52. 10.1002/jsfa.8462 28580581

[49] Azizan A Xin LA Abdul Hamid NA Maulidiani M Mediani A Abdul Ghafar SZ Potentially bioactive metabolites from pineapple waste extracts and their antioxidant and α-glucosidase inhibitory activities by ^1^H NMR. Foods. 2020;9(2):173. 10.3390/foods9020173 32053982 PMC7073707

[50] Alam MA Zaidul ISM Ghafoor K Sahena F Hakim MA Rafii MY *In vitro* antioxidant and α-glucosidase inhibitory activities and comprehensive metabolite profiling of methanol extract and its fractions from *Clinacanthus nutans.* BMC Complement Altern Med. 2017;17(1):181–91. 10.1186/s12906-017-1684-5 28359331 PMC5374668

[51] Venkatachalam R Kalimuthu K Chinnadurai V Saravanan M Radhakrishnan R Shanmuganathan R Various solvent effects on phytochemical constituent profiles, analysis of antioxidant and anti-diabetic activities of *Hopea parviflora.* Process Biochem. 2019;89:227–32. 10.1016/j.procbio.2019.10.025

[52] Oon SF Nallappan M Tee TT Shohaimi S Kassim NK Sa’ariwijaya MSF Xanthorrhizol: A review of its pharmacological activities and anti-cancer properties. Cancer Cell Int. 2015;15:100–15. 10.1186/s12935-015-0255-4 26500452 PMC4618344

[53] Choi JS Islam MN Ali MY Kim YM Park HY Sohn HS The effects of C-glycosylation of luteolin on its antioxidant, anti-Alzheimer’s disease, anti-diabetic, and anti-inflammatory activities. Arch Pharm Res. 2014;37(10):1354–63. 10.1007/s12272-014-0351-3 24988985

[54] Kalaycıoğlu Z Gazioğlu I Erim FB. Comparison of antioxidant, anticholinesterase, and anti-diabetic activities of three curcuminoids isolated from *Curcuma longa* L. Nat Prod Res. 2017;31(24):2914–7. 10.1080/14786419.2017.1299727 28287280

